# Experimental Investigation on Factors Influencing the Early-Age Strength of Geopolymer Paste, Mortar, and Concrete

**DOI:** 10.3390/ma18245648

**Published:** 2025-12-16

**Authors:** Shiyu Yang, Jamal A. Abdalla, Rami A. Hawileh, Jianhua Liu, Yaqin Yu, Zhigang Zhang

**Affiliations:** 1 College of Civil and Construction Engineering, Hunan Institute of Technology, Hengyang 421002, China; shyouyang@hnit.edu.cn (S.Y.);; 2 School of Civil Engineering, Southwest Jiaotong University, Chengdu 610031, China; 3 Department of Civil Engineering, American University of Sharjah, Sharjah P.O. Box 26666, United Arab Emirates; jabdalla@aus.edu (J.A.A.); rhaweeleh@aus.edu (R.A.H.); 4 Energy, Water, and Sustainable Environment Research Center, American University of Sharjah, Sharjah P.O. Box 26666, United Arab Emirates; 5 School of Civil Engineering, Chongqing University, Chongqing 400045, China; 6 State Key Laboratory of Safety and Resilience of Civil Engineering in Mountain Area, Chongqing University, Chongqing 400045, China

**Keywords:** geopolymer, mechanical properties, microstructure, alkaline activator, predictive model

## Abstract

This study systematically investigates the key parameters governing the mechanical performance of fly ash-based geopolymer across paste, mortar, and concrete scales. Comprehensive mechanical testing, combined with SEM and MIP analyses, elucidated the relationships between activator composition, pore structure, and strength development. A key innovation is the development of a cross-scale quantitative framework linking mortar strength to concrete compressive strength, enabling preliminary predictive capability across material scales. Grey relational analysis identified curing temperature as the most influential factor, followed by SiO_2_/Na_2_O and H_2_O/Na_2_O ratios. Thermal curing accelerates strength development and temperatures of 70~80 °C markedly enhance reaction rates. Both compressive and flexural/splitting tensile strengths increase and then decrease with NaOH concentration or sodium silicate modulus, with optimal performance at 24~26% NaOH and SiO_2_/Na_2_O ratio of 1.2~1.4, while increasing H_2_O/Na_2_O reduces strength nearly linearly, constrained by workability. Concrete compressive strength rises with coarse aggregate content up to 60~70% before declining. SEM and MIP confirm that optimal activator formulations produce a dense, homogeneous gel matrix with lower porosity and fewer unreacted particles. Strong square-root correlations between compressive and tensile-related strengths were observed across all material systems. Overall, this work establishes a quantitative foundation for geopolymer mix design and provides actionable guidance for developing high-performance, low-carbon geopolymer concrete.

## 1. Introduction

In recent years, the rapid expansion of global infrastructure has led to a substantial increase in the consumption of Portland cement. The production of this essential building material is energy-intensive and accounts for approximately 7~8% of global anthropogenic carbon dioxide (CO_2_) emissions, prompting a critical need for sustainable and environmentally friendly alternatives [1]. To improve the greenness of concrete, the supplementary cementitious materials (SCMs), such as fly ash, rice husk ash, and waste steel slag, were incorporated to partially substitute cement [2,3,4,5]; alternatively, some industrial wastes were used in place of aggregates [6,7,8,9]. In addition, geopolymer binders synthesized from industrial by-products, such as fly ash (FA) and ground granulated blast furnace slag (GGBFS), have garnered significant attention. These materials offer a promising pathway to reducing the carbon footprint of the construction industry due to their high strength, excellent durability, and significantly lower environmental impact.

Geopolymers are inorganic cementitious materials that can partially or completely replace ordinary Portland cement (OPC). They are formed by the alkaline activation of source materials rich in silica (SiO_2_) and alumina (Al_2_O_3_). Microscopically, the geopolymer structure consists of a three-dimensional network of corner-sharing [SiO_4_]^4−^ and [AlO_4_]^5−^ tetrahedral, linked by oxygen atoms to form an –Si–O–Al– framework [10]. This unique alumina-silicate network structure imparts geopolymer with superior mechanical properties and durability, including notable resistance to frost [11], low permeability [12], high-temperature stability [13], and the ability to immobilize heavy metals [14]. Due to the abundance of raw materials, simple production process, low energy consumption, and reduced CO_2_ emissions, geopolymer technology is regarded as a cornerstone for developing “green concrete” and has attracted increasing research interest.

The concept of “alkali activation” was first introduced by Purdon in Belgium while studying the hardening mechanism of OPC. Subsequently, in the 1960s, Glukhovsky from the former Soviet Union proposed the “alkaline reaction mechanism” based on extensive research [15]. The field was significantly advanced by the French scientist Davidovits, who, in 1978, synthesized a novel cementitious material by activating metakaolin (MK) with a strong alkali and termed it a “geopolymer” [16]. Since then, research into geopolymer chemistry, properties, and applications has become a prominent topic in materials science [17,18,19].

The performance of geopolymer concrete is strongly influenced by the characteristics of the source materials, including chemical composition, particle size, and the content of reactive components [20,21,22,23]. Even materials with similar nominal compositions can exhibit significant differences in mechanical performance, often due to variations in the proportion of reactive silicon and aluminum species [24]. Particle size distribution critically affects dissolution kinetics and microstructural development in geopolymer. Assi et al. [25] reported that finer particle distributions enhance compressive strength; for instance, reducing the average fly ash particle size from 4.78 μm to 4.78 μm increased the compressive strength of geopolymer by 210%. In contrast, Soutsos et al. [26] found that a higher cumulative volume of particles larger than 45 μm, combined with a smaller average particle size, led to increased compressive strength, likely by optimizing packing density and microstructural connectivity.

Alkaline activators, typically a mixture of sodium hydroxide (NaOH) and sodium silicate (Na_2_SiO_3_), further govern the development of geopolymer strength. The NaOH concentration drives the dissolution of aluminosilicate species, while the SiO_2_/Na_2_O ratio controls the resulting gel network and microstructure [27,28]. However, reported optimal activator formulations vary widely. For instance, Somna et al. [29] found that a water glass to NaOH ratio of 0.7 yielded maximum compressive strength for fly ash-based geopolymers, whereas Rittaruth et al. [30] recommended a ratio of 1.5. For single-component activators, Komljenovic et al. [31] reported that NaOH was more effective than KOH, a finding contradicted by Jiao et al. [32]. Moreover, independent studies by Görhan et al. [33] and Sukmak et al. [34] identified optimal NaOH concentrations of 6 mol/L and 14 mol/L, respectively. These discrepancies underscore the sensitivity of geopolymer strength to activator chemistry and highlight the need for systematic, quantitative investigations to define optimal formulations, particularly for early-age performance.

Curing conditions, including method, temperature, and humidity, have also been shown to significantly impact the properties of the final product in geopolymer [35,36]. Al-majidi et al. [37] observed that heat curing had no effect on the 28-day compressive strength of a fly ash/slag-based geopolymer. In contrast, Heah et al. [38] reported a fourfold increase in the compressive strength of metakaolin-based geopolymer with high-temperature curing. Meanwhile, Rovnanik [39] concluded that low-temperature curing produced a denser and more uniform microstructure. These discrepancies indicate that qualitative descriptions of activator composition cannot adequately represent the underlying reaction chemistry.

Despite extensive research, no consensus has been reached regarding the key parameters governing the compressive strength of geopolymer, and a universally accepted mix-design methodology remains unavailable. The large variability reported in the literature is partly attributable to differences in the chemical composition of source materials, but more fundamentally to the absence of a unified framework capable of quantitatively describing how activator chemistry controls strength development across paste, mortar, and concrete scales. Most existing studies employ empirical mass or volume ratios, or nominal silicate moduli, which do not accurately represent the chemical contributions of Na_2_O, SiO_2_, and H_2_O in the NaOH–Na_2_SiO_3_ system. As a consequence, the roles of these components in dissolution, gel formation, and strength evolution cannot be quantitatively compared across studies, limiting reproducibility and generalizability.

Furthermore, the recent surge in applications such as precast production, 3D printing, rapid construction, and fiber-reinforced composites places particular emphasis on early-age (hours to days) strength development [40], which governs setting kinetics, buildability, and structural integrity. However, most existing research focuses on 28-day strength, leading to insufficient understanding of early-age behavior and its relationship to activator chemistry. To address these gaps, this study reformulates composite activators into a unified Na_2_O·*n*SiO_2_·*z*H_2_O system. Here, *n* represents the molar ratio of SiO_2_ to Na_2_O, and *z* denotes the mass ratio of total water to Na_2_O, including the water contained in the liquid Na_2_SiO_3_ and any additional free water. This approach allows for an independent control and quantitative assessment of (i) the SiO_2_/Na_2_O ratio (*n*), which governs silicate speciation and gel composition, and (ii) the H_2_O/Na_2_O ratio (*z*), which dictates alkalinity, OH^−^ activity, and dissolution potential. This framework enables direct comparison across different formulations and provides a basis for evaluating how activator chemistry drives pore refinement, reaction extent, and early-age strength development.

## 2. Materials and Methods

### 2.1. Source Materials

**Figure 1:**
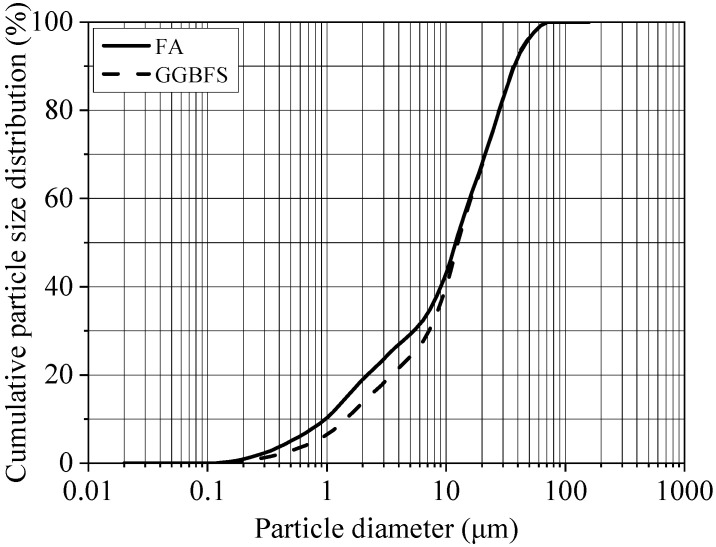
Particle size distribution of FA and GGBFS.

The study employed two primary binders: a low-calcium Class F fly ash (FA) compliant with ASTM C618 [41], supplied by Chengdu Bolei Resource Recycling Development Co., Ltd., Chengdu, China; and a ground granulated blast-furnace slag (GGBFS) meeting the ASTM C989 Grade 95 [42] specification, provided by Chengdu Concrete New Materials Co., Ltd., Chengdu, China. The chemical compositions of the FA and GGBFS, determined by X-ray fluorescence (XRF), are presented in Table 1. The particle size distributions were measured using a laser diffraction analyzer, as shown in Figure 1.

As detailed in Table 1, the combined content of SiO_2_ and Al_2_O_3_ in the FA was 84.5%, providing an abundant source of precursors for geopolymerization. The GGBFS was characterized by a high CaO content of 35.07%, which is beneficial for enhancing the early-age strength of the geopolymer. Figure 1 illustrates that the particle size distributions of FA and GGBFS are similar, with particle diameters ranging from 0.1 to 100 μm. The cumulative volume fractions of particles smaller than 45 μm were 94.26% for FA and 94.56% for GGBFS, with median particle sizes (D_50_) of 12.1 μm and 12.5 μm, respectively.

**Figure 2:**
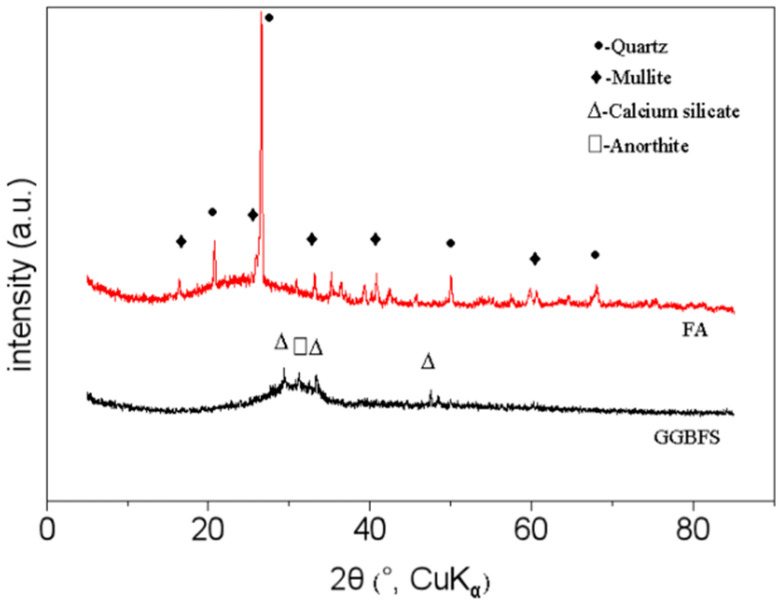
The XRD pattern of FA and GGBFS.

The mineralogical compositions of the FA and GGBFS were analyzed by X-ray diffraction (XRD), with the results shown in Figure 2. The XRD pattern of the FA reveals a broad amorphous halo between 15° and 35° (2θ), indicating a high content of reactive glassy phases. Superimposed on this halo are sharp diffraction peaks corresponding to crystalline phases, primarily identified as quartz (at 2θ = 21.0°, 26.6°, and 65.0°) and mullite (at 2θ = 17.1° and 28.3°). In contrast, the XRD pattern of the GGBFS is predominantly amorphous, characterized by a broad hump between 15° and 35° (2θ) and only minor crystalline peaks. These results confirm that both FA and GGBFS possess substantial amorphous content, making them suitable precursors for geopolymer synthesis.

### 2.2. Aggregates and Alkaline Activator

A mixture of sodium hydroxide solution and sodium silicate (water glass) was employed as the alkaline activator. Analytical-grade sodium hydroxide pellets (≥98% purity) were supplied by Chengdu Cologne Chemical Co., Ltd., Chengdu, China. The analytical-grade sodium silicate solution, obtained from Guangdong Foshan Kening New Materials Technology Co., Ltd., Foshan, China, had a density of 1.535 g/cm^3^ and a silica modulus (SiO_2_/Na_2_O) of 2.43, with a chemical composition of 13.73 wt% Na_2_O, 32.35 wt% SiO_2_, and 53.92 wt% H_2_O. Mortar specimens were prepared using ISO standard sand in accordance with GB/T 17671-2021 [43]. For concrete mixtures, the fine aggregate was quartz sand supplied by Chengdu Water New Materials Technology Co., Ltd., Chengdu, China, with a fineness modulus of 2.81. The coarse aggregate consisted of graded crushed stone with particle sizes ranging from 5.0 to 20.0 mm, sourced from local suppliers in Chengdu, Sichuan, China. Its physical properties satisfied the requirements specified in GB/T 14685-2022 [44].

### 2.3. Specimen Preparation and Curing

All samples were prepared following standardized procedures to maintain specimen uniformity. Specifically, the composite activator solution (NaOH, liquid Na_2_SiO_3_, and additional water) was prepared 24 h in advance and allowed to cool to room temperature to achieve thermal and ionic equilibrium. For mortar mixtures, standard sand and the binders (FA and GGBFS) were placed in the mixing pan of a pre-moistened planetary mortar mixer (manufactured by Jianding Construction Instrument Factory, Wuxi, China) and mixed at low speed (140 ± 5 rpm) for 30 s, during which the activator solution was gradually added. The mixer was then switched to high speed (285 ± 10 rpm) for 30 s, while the standard sand was evenly added. Finally, the mixture was alternately stirred at high and low speeds for 60 s each to ensure uniformity. The prepared mortar was cast into 40 mm × 40 mm × 160 mm prismatic molds. The procedure for paste specimens was similar, except that no fine aggregate was used. The concrete mixtures were prepared using an SJD60 single-shaft compulsory mixer (60 L capacity) manufactured by Shanghai Dongxing Construction Material Testing Equipment Co., Ltd., Shanghai, China. The pre-weighed aggregates were first placed in the slightly moist but free-of-pool-water mixer and mixed at 45 rpm for 2 min. FA and GGBFS were then added and mixed for another 2 min. Finally, the activator solution (including water) was slowly and evenly poured into the mixer during mixing for 3 min. The resulting concrete mixture was cast into 100 mm × 100 mm × 100 mm cubic molds.

**Figure 3:**
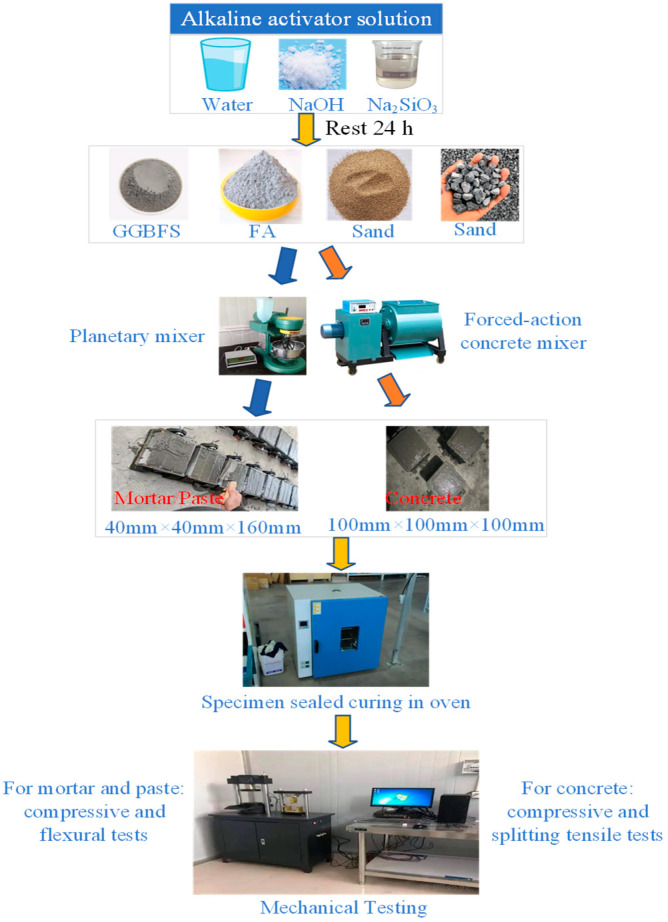
Key steps in the preparation of concrete/mortar/paste.

After casting, all specimens were pre-cured at a controlled laboratory temperature of 25 ± 1 °C for 24 h. They were then demolded, sealed with linear polyethylene film to prevent moisture loss, and subjected to heat curing at 80 °C for 24 h (except for specimens used to study the effect of curing temperature or duration). After heat curing, the specimens were returned to the laboratory at 25 ± 1 °C, covered with film, and stored until testing. The key steps of the preparation process are illustrated in Figure 3.

### 2.4. Mix Proportion and Testing Methods

Based on the preliminary tests, the detailed mix proportions are listed in Table 2. The experimental program was designed to investigate the effects of FA particle size, NaOH concentration, SiO_2_/Na_2_O ratio (*n*), H_2_O/Na_2_O ratio (*z*), curing temperature, curing duration, solution/binder ratio, binder/sand ratio, and GGBFS content on the mechanical properties of geopolymer paste, mortar, and concrete. The influencing factors investigated using the single-factor method are summarized in Table 3.

The mechanical properties of the mortar and paste were evaluated following the procedures specified in GB/T 17671-2021 [43]. All tests were conducted using a WDW-Y300D microcomputer-controlled testing machine (300 kN capacity) manufactured by Jinan Xingguang Testing Machine Co., Ltd., Jinan, China. Specifically, the flexural strength was tested on 40 mm × 40 mm × 160 mm prismatic specimens loaded at mid-span at a rate of 50 ± 10 N/s until failure, with the reported value being the average of three specimens. The compressive strength was determined on the six resultant half-prisms (effective 40 mm cubes) from the flexural test, loaded at a rate of 2400 ± 200 N/s until failure, with the result averaged from these six specimens. The mechanical properties of concrete were tested following the Chinese national standard GB/T 50081-2019 [45]. Specifically, The mechanical properties of the concrete were evaluated using a YAW-1000 electro-hydraulic servo testing machine (maximum capacity: 1000 kN), produced by SANS Zhongheng, Shenzhen, China. Cubic specimens with side length of 100 mm were used, with compressive strength measured at a loading rate of 0.6 MPa/s. The tensile strength of the concrete was also evaluated using the splitting test on 100 mm cubic specimens. In this method, the specimen is loaded along one axis until failure, and the resulting load is used to calculate the tensile strength. All reported strength values represent the average of three replicate specimens.

### 2.5. Microstructural Characterization

The microstructure and pore structure of selected hardened mortar specimens were analyzed to understand the strength development mechanisms. The microstructural morphology was examined using a KYKY EM3200 scanning electron microscope (SEM) manufactured by Beijing Zhongke Instrument Co., Ltd., Beijing, China. Small fragments obtained from the core of the specimens were dried in an oven and then sputter-coated with a thin layer of platinum prior to imaging. Porosity and pore size distribution were measured using mercury intrusion porosimetry (MIP) with a Micromeritics AutoPore IV 9500 instrument (Norcross, GA, USA). Prior to testing, hardened samples were immersed in absolute ethanol to stop the hydration process and then dried at 60 °C to a constant mass. The analysis was conducted over a pressure range from 0.01 to 200 MPa.

### 2.6. Grey Relational Analysis

Grey relational analysis (GRA) was employed to quantitatively evaluate the influence of multiple factors on the mechanical properties. The calculation procedure is as follows:

(1) Determination of sequences

The reference sequence (e.g., compressive strength) and comparison sequences (influencing factors) are determined.

(2) Calculate the mean value of each sequence $\bar{x}(k)$

(1) $$\bar{x}(k)=\frac{1}{n}\sum_{n}^{k=1}x(k)$$

Here, *k* denotes the position of a value in the parent sequence; *x*(*k*) represents the value of the sequence; and *n* is the number of values in the parent sequence.

(3) Normalize each sequence to obtain dimensionless standardized data sequences *x*′(*k*)

(2) $$x^{\prime }(k)=\frac{x(k)}{\bar{x}(k)};k=1,2,\ldots ,n$$

(4) Calculate the absolute difference (Δ*_ci_*(*k*)) between the elements of each comparison child sequence (x′_0_(*k*)) and the corresponding elements of the parent sequence (x′*_i_*(*k*))

(3) $$\Delta_{ci}(k)=\left|x_{0}^{\prime }(k)-x_{i}^{\prime }(k)\right|;\;\;\;\;k=1,2\ldots ,n;i=1,2,\ldots ,m$$

(5) Determine the maximum (*M*) and minimum (*q*) of the absolute differences

(4) $$M=\underset{i}{\max}\underset{k}{\max}\Delta_{ci}(k)$$

(5) $$q=\underset{i}{\min}\underset{k}{\min}\Delta_{ci}(k)$$

(6) Calculate the correlation coefficient between each child sequence and the parent sequence *L_ci_*(*k*)

(6) $$L_{c_{i}}(k)=\frac{q+\xi M}{\Delta_{i}(k)+\xi M}$$

Here, *ξ* is the resolution coefficient, with ∈ (0, 1) and is typically taken as 0.5.

(7) Calculate the degree of correlation (*γ_ci_*) between each child sequence and the parent sequence

(7) $$\gamma_{ci}=\frac{1}{n}\sum L_{ci}(k)$$

A higher grey relational degree (*γ_ci_*) indicates a stronger influence of the corresponding parameter on the parent sequence, reflecting its greater importance.

## 3. Results and Discussion

### 3.1. Effect of FA Particle Size on Mechanical Strength

**Figure 4:**
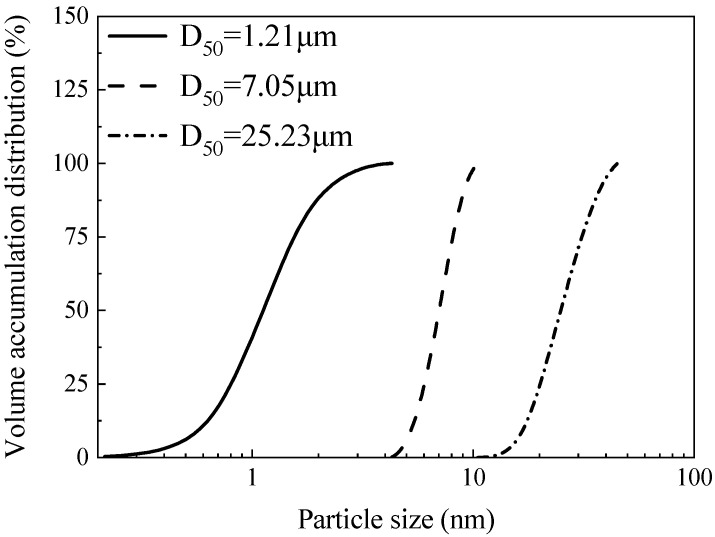
Particle size distribution of FA after fine grinding and negative-pressure separation.

To evaluate the influence of precursor particle size, the FA was mechanically ground and sieved into three distinct size fractions: <4.3 μm, 4.3–10.5 μm, and 10.5–45 μm. The particle size distributions for these fractions are detailed in Figure 4.

**Figure 5:**
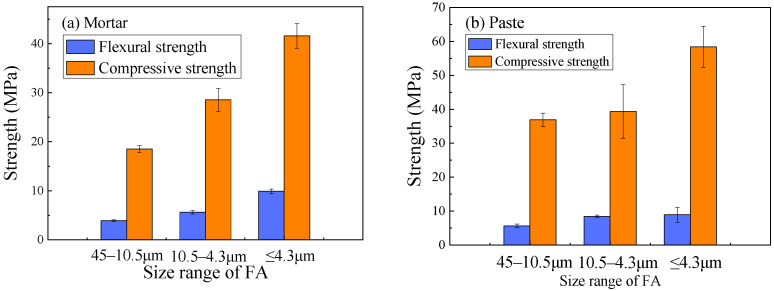
Effect of FA particle size on the mechanical strength of geopolymer mixtures.

The mechanical properties of the resulting geopolymer specimens were strongly dependent on the FA particle size, as shown in Figure 5. For geopolymer mortar, the compressive strength decreased from 41.58 MPa to 28.52 MPa and further to 18.53 MPa as the FA particle size range increased from <4.3 μm to 4.3~10.5 μm and 10.5~45 μm, respectively. A similar trend was observed for the compressive strength of the paste and the flexural strength of the mortar.

This phenomenon can be attributed to the specific surface area and reactivity of the FA particles. Finer particles possess a significantly larger surface area, which accelerates their dissolution in the highly alkaline environment of the activator solution. This enhanced dissolution provides a greater concentration of reactive silicate and aluminate species, facilitating a more extensive and complete geopolymerization reaction. Consequently, a denser, more homogeneous, and stronger geopolymer matrix is formed, leading to superior mechanical performance.

### 3.2. Effect of NaOH Concentration

**Figure 6:**
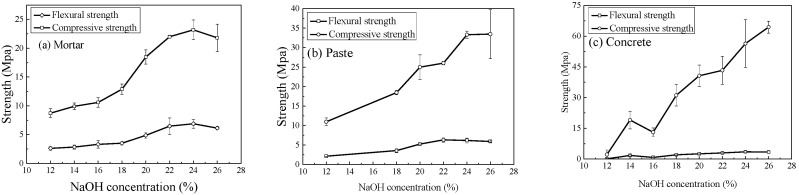
Effect of NaOH concentration on the strength of geopolymer specimens.

The concentration of the NaOH solution plays a critical role during the activation process. As shown in Figure 6, when the NaOH concentration increases from 12% (3.39 M) to 24% (7.61 M), the compressive strength of the geopolymer mortar increases by 166.0%. However, a further increase in concentration leads to a slight decline, indicating that 24% (7.61 M) is the optimal concentration. Similarly, increasing the NaOH concentration from 12% (3.39 M) to 24% (7.61 M) results in a 233.0% improvement in the compressive strength of the geopolymer paste, while additional increases cause no significant change. The flexural strength of the geopolymer mortar and paste exhibits a trend consistent with that of compressive strength. In contrast, both the compressive strength and splitting tensile strength of geopolymer concrete continue to increase with rising NaOH concentration. When the concentration is raised from 12% (3.39 M) to 26% (8.40 M), the compressive strength of the concrete increases by approximately 27-fold, and the splitting tensile strength increases by 28.5-fold. In this study, the maximum NaOH concentration examined was 26% (8.40 M), as it approaches the saturation limit at room temperature (25 °C).

The dependence of mechanical strength on NaOH concentration arises from the interplay between hydroxyl-ion activity and silicate chemistry. At low to moderate concentrations, increased OH^−^ activity promotes rapid dissolution of aluminosilicate phases, supplying abundant soluble Si and Al for geopolymer gel formation. This enhances polycondensation, increases gel cross-linking, and produces a denser microstructure, thereby improving compressive and flexural strengths. At near-saturation concentrations, however, excess Na^+^ begins to adsorb on particle surfaces and disrupt network growth, while changes in silicate speciation and solution viscosity restrict the mobility of reactive species. These effects limit gel densification and reduce the efficiency of polymerization, resulting in an optimum strength at intermediate NaOH concentrations followed by a plateau or decline at higher levels. The SEM and MIP microstructure observations in Section 3.8 confirm this mechanism.

### 3.3. Effect of Sodium Silicate Activator

#### 3.3.1. Effect of SiO_2_/Na_2_O Ratio

**Figure 7:**
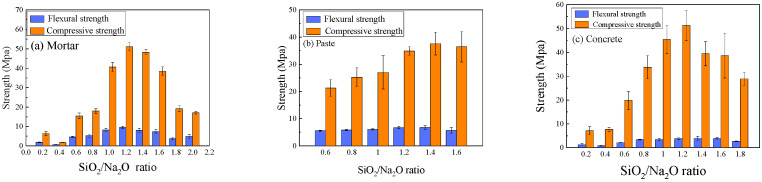
Effect of SiO_2_/Na_2_O ratio on the strength of geopolymer specimens.

The SiO_2_/Na_2_O ratio (referred to as modulus, *n*) of the activator solution significantly influenced the mechanical properties of all geopolymer mixtures, as depicted in Figure 7. The compressive, flexural, and splitting tensile strengths of the paste, mortar, and concrete specimens all exhibited a similar parabolic trend with increasing SiO_2_/Na_2_O ratio. The mechanical properties improved as SiO_2_/Na_2_O ratio increased from 0.2, reaching a peak value when SiO_2_/Na_2_O ratio was in the range of 1.2 to 1.4. Further increasing the SiO_2_/Na_2_O ratio beyond this optimal range resulted in a progressive reduction in strength.

This behavior underscores the importance of balancing the dissolution and polycondensation stages of geopolymerization. The Na_2_O component governs the alkalinity, which is essential for dissolving the solid precursors. The SiO_2_ component provides soluble silicate species that act as the primary building blocks for the geopolymer network. At a low SiO_2_/Na_2_O ratio, the system has high alkalinity but insufficient silicate species, limiting the extent of polycondensation. As the SiO_2_/Na_2_O ratio increases towards the optimal range, a favorable balance is achieved, promoting both efficient dissolution and extensive network formation, resulting in a dense microstructure and high strength. However, when the SiO_2_/Na_2_O ratio becomes too high (*n* > 1.4), the viscosity of the sodium silicate solution increases sharply. This high viscosity not only hinders the mobility of ions but also traps air bubbles introduced during mixing. These entrapped bubbles create pores and defects within the hardened matrix, thereby compromising the material’s strength.

#### 3.3.2. Effect of H_2_O/Na_2_O Ratio

**Figure 8:**
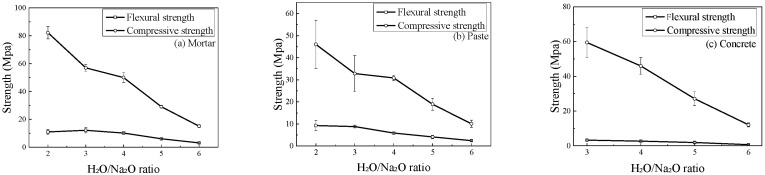
Effect of H_2_O/Na_2_O ratio on the strength of geopolymer specimens.

The H_2_O/Na_2_O ratio (*z*), which represents the water content in the activator, had a pronounced and near-linear negative effect on the mechanical properties of all geopolymer mixtures, as shown in Figure 8. As *z* increased from 2.0 to 6.0, the compressive strength of the mortar specimens decreased sharply from 82.2 MPa to 15.4 MPa. Similar significant reductions in compressive and flexural strengths were observed for the mortar and paste specimens. 

Water plays a crucial role as a medium for ion transport during the geopolymerization reaction, but excess water is detrimental to the final strength. A lower *z* value corresponds to a higher concentration of the alkaline activator, which accelerates the dissolution of FA and increases the concentration of silicate oligomers. This promotes the formation of a denser, more cross-linked geopolymer network. Conversely, a higher water content dilutes the activator, slowing the reaction kinetics. Furthermore, the excess water that does not participate in the reaction remains as pore water within the matrix, eventually evaporating to leave behind a more porous microstructure. This increased porosity directly contributes to the reduction in mechanical strength. While lower water content is beneficial for strength, a practical lower limit of *z* = 3.0 is necessary to ensure adequate workability for proper mixing and casting of the concrete.

### 3.4. Effect of Curing Conditions and Failure Modes

#### 3.4.1. Effect of Curing Temperature and Duration

**Figure 9:**
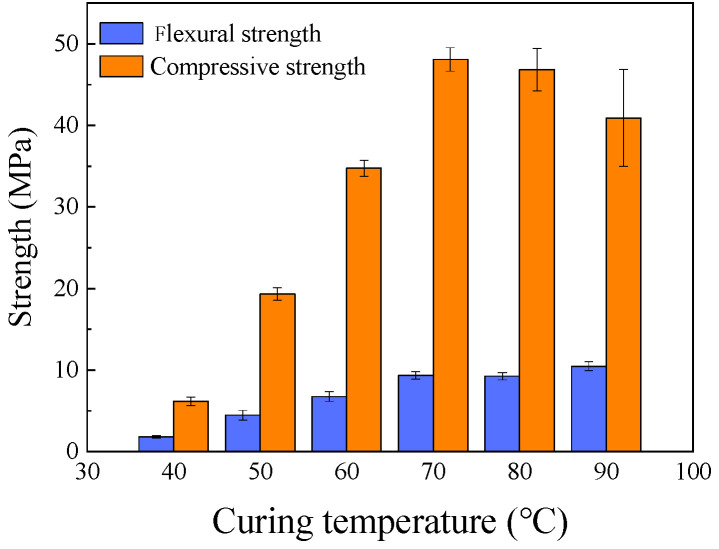
Effect of curing temperature on mortar strength.

Curing temperature is a critical parameter for the strength development of FA-based geopolymers. As shown in Figure 9, the compressive and flexural strengths of the mortar increased significantly and almost linearly as the curing temperature was raised from 40 °C to 70 °C. The compressive strength, for instance, increased from 6.17 MPa at 40 °C to 48.08 MPa at 70 °C. This indicates that thermal energy is essential to accelerate the dissolution of the relatively low-reactivity FA. When the temperature was further increased from 70 °C to 90 °C, the compressive strength showed only marginal improvement, while the flexural strength slightly decreased. This suggests that excessively high temperatures can lead to rapid water evaporation from the specimen surface, creating internal stresses and microcracks that are particularly detrimental to properties governed by tensile strength, such as flexural strength.

**Figure 10:**
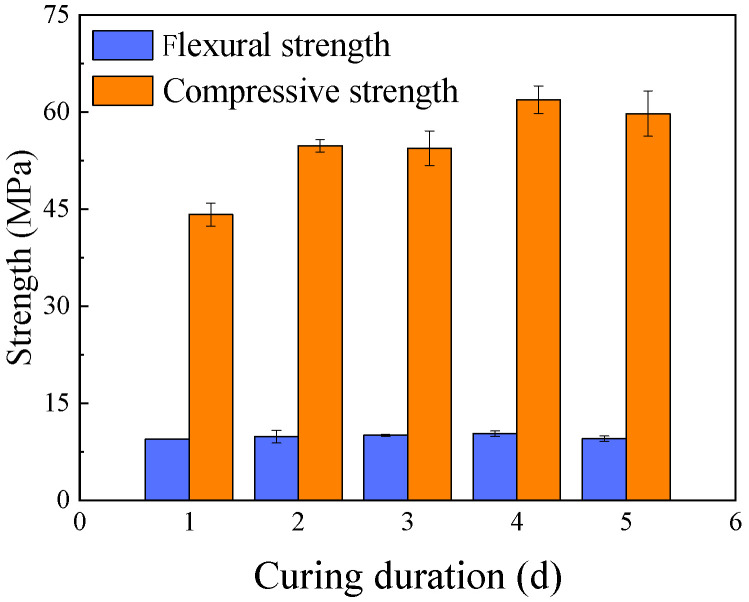
Effect of curing duration on mortar strength.

The effect of curing duration at an elevated temperature is presented in Figure 10. The results show that the majority of the strength gain occurred within the first 24 h of curing, reaching 71.3% of the maximum compressive strength and 91.9% of the maximum flexural strength. Extending the curing duration to 4 days resulted in the peak strength, after which a slight decrease was observed. This indicates that prolonged heat curing provides diminishing returns and is not cost-effective. A curing duration of 1 day appears to be sufficient for achieving a substantial degree of geopolymerization and strength development.

#### 3.4.2. Failure Modes of Geopolymer Specimens

**Figure 11:**
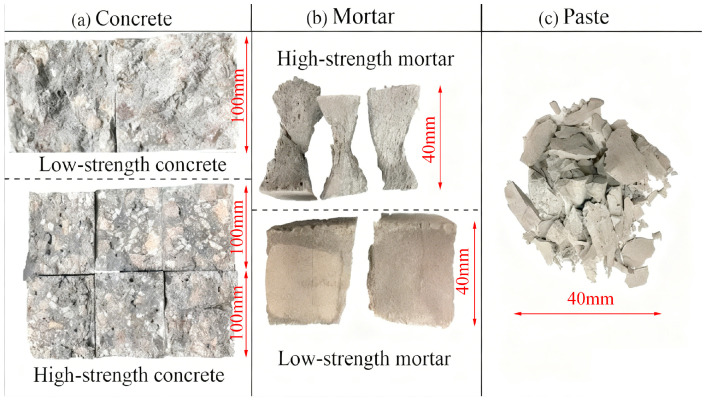
Failure modes of geopolymer specimens. Note: Failure patterns of the concrete specimens correspond to splitting-tensile loading, whereas those of the mortar and paste correspond to compressive loading.

The failure modes of the geopolymer specimens under mechanical loading were observed to be similar to those of conventional Portland cement composites, exhibiting characteristic brittle failure, as shown in Figure 11. For low-strength concrete, the fracture plane tended to follow the weaker interface between the aggregates and the geopolymer paste (Figure 11a, top). In contrast, high-strength concrete specimens exhibited trans-granular fracture, where the crack propagated through both the paste and the coarse aggregates, resulting in a smoother fracture surface (Figure 11a, bottom).

High-strength mortar specimens under compression failed in a conical shape, a result of the confining pressure (ring hoop effect) from the testing machine platens at the ends, while the unconfined middle section expanded laterally (Figure 11b, top). Low-strength mortar developed several vertical microcracks before one crack propagated rapidly, leading to failure (Figure 11b, bottom). The paste specimens typically shattered into many needle-like fragments upon failure (Figure 11c).

### 3.5. Effect of Mix Proportion Ratios

#### 3.5.1. Effect of Solution/Binder Ratio

**Figure 12:**
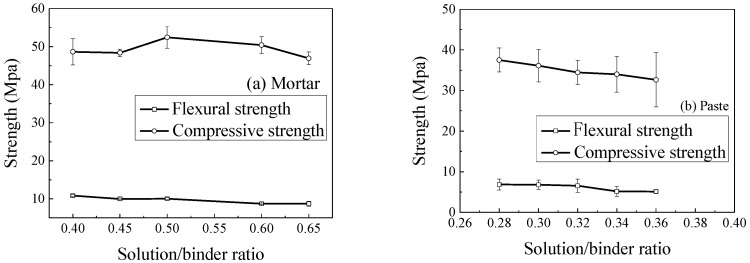
Effect of solution/binder ratio on specimen strength.

The solution/binder ratio showed a negative correlation with the mechanical strength of the geopolymer mixtures, analogous to the effect of the water/cement ratio in traditional concrete (Figure 12). For instance, as the solution/binder ratio for mortar increased from 0.4 to 0.65, the flexural strength decreased by 19.9% (from 10.83 MPa to 8.70 MPa), while the compressive strength showed a more modest decline. A higher solution/binder ratio introduces more water into the system, leading to higher porosity in the hardened matrix and consequently lower strength. Therefore, to achieve high performance, the lowest possible solution/binder ratio that still provides adequate workability should be selected.

#### 3.5.2. Effect of Binder/Sand Ratio

**Figure 13:**
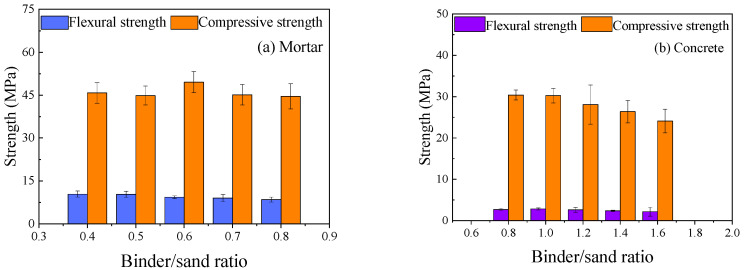
Effect of binder/sand ratio on specimen strength.

The influence of the binder/sand ratio on the mechanical properties is presented in Figure 13. For mortar, the compressive strength remained relatively stable at around 45 MPa as the binder/sand ratio increased from 0.4 to 0.8. However, the flexural strength decreased by 18.3% (from 10.4 MPa to 8.5 MPa). This reduction in flexural strength may be attributed to an increase in the thickness of the paste layer surrounding the sand particles at higher binder contents, which can be detrimental to the tensile load-bearing capacity of the mortar. For concrete, a similar trend was observed, with strength values declining when the binder/sand ratio exceeded 1.2. These results suggest that an optimal binder/sand ratio exists and that a lower ratio is generally preferable for designing mix proportions, provided that workability is maintained.

### 3.6. Grey Relational Analysis of Influencing Factors

The calculated grey relational degrees are summarized in Table 4, and the analysis reveals the hierarchy of factors influencing the geopolymer’s strength. For compressive strength, the factors are ranked in descending order of importance as follows: curing temperature (*T*) > SiO_2_/Na_2_O ratio (*n*) > H_2_O/Na_2_O ratio (*z*) > solution-to-binder ratio (*S*) > binder-to-sand ratio (*C*) > curing duration (*t*) > GGBFS content (*ω*). The ranking for flexural strength is slightly different: *T* > *n* > *z* > *C* > *S* > *t* > *ω*. These results clearly indicate that curing temperature and the chemical composition of the activator (*n* and *z*) are the most dominant factors controlling the strength development of the geopolymer system. In contrast, the GGBFS content had the least significant impact within the range studied.

### 3.7. Correlation Between Compressive and Flexural Strength

**Figure 14:**
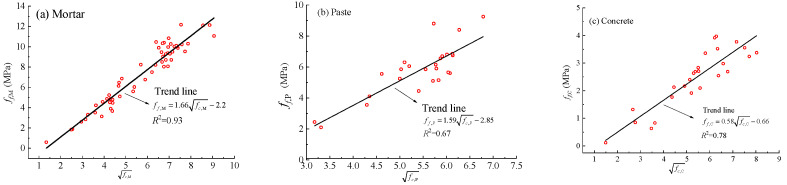
Relationship between compressive strength and flexural strength of geopolymer mixtures.

The square-root type correlation between compressive strength and flexural (or splitting tensile) strength are essential for material design and structural analysis. Based on the experimental data obtained in this study, regression analyses were performed. As shown in Figure 14, a strong square-root correlation was identified between the compressive strength (*f_c_*_,*M*_) and flexural strength (*f_f_*_,*M*_) of the geopolymer mortar, which can be expressed as follows:

(8) $$f_{f,M}=1.66\sqrt{f_{c,M}}-2.20,\begin{array}{cc} & R^{2}\;=\;0.93 \end{array}$$

Similarly, the regression relationships between compressive strengths (*f_c_*_,*P*_) and flexural strength (*f_f_*_,*p*_) for the geopolymer paste, and between compressive strength (*f_c_*_,*C*_) and splitting tensile (*f_f_*_,*C*_) strength for the geopolymer concrete, are presented in Equations (9) and (10), respectively.

(9) $$f_{f,P}=1.59\sqrt{f_{c,P}}-2.85,\begin{array}{cc} & R^{2}=0.67 \end{array}$$

(10) $$f_{f,C}=0.58\sqrt{f_{c,C}}-0.66,\begin{array}{cc} & R^{2}=0.78 \end{array}$$

These relationships resemble those observed in conventional cement-based materials, reflecting a similar underlying dependence of tensile-type strengths on compressive capacity within geopolymer matrices.

### 3.8. Microstructural Analysis

To elucidate the underlying mechanisms governing the mechanical properties, the microstructure and pore structure of representative geopolymer mortar specimens were characterized using SEM and MIP.

#### 3.8.1. SEM Analysis

**Figure 15:**
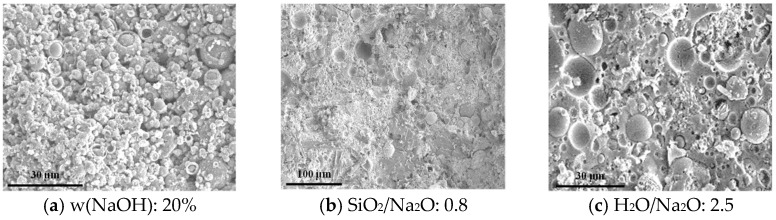

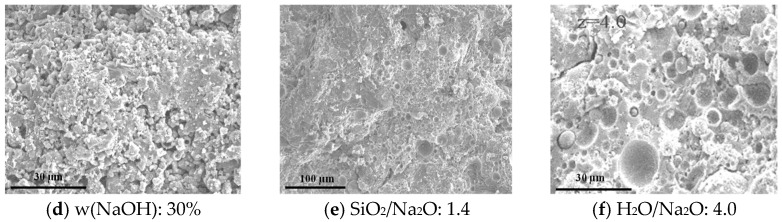
Microscopic appearance of geopolymer mortar.

The SEM micrographs in Figure 15 reveal the influence of activator composition on the geopolymer matrix morphology. At a low NaOH concentration (20%), the microstructure was loose and porous, with many unreacted or partially dissolved FA particles visible. This incomplete reaction explains the low compressive strength observed in these specimens. Increasing the NaOH concentration to 30% resulted in a significantly denser matrix with fewer residual FA spheres, indicating a more extensive dissolution and reaction.

When a composite activator was used, the resulting geopolymer matrix was notably denser and more homogeneous. A comparison between specimens with different SiO_2_/Na_2_O ratios (*n*) showed that while the extent of FA dissolution was similar, the specimen with *n* = 0.8 (closer to the optimal value for strength) exhibited a more continuous and uniform geopolymer gel compared to the specimen with *n* = 1.4. This suggests that an optimal SiO_2_/Na_2_O ratio promotes not just dissolution but also more efficient polycondensation.

The effect of water content is also evident. Both specimens with H_2_O/Na_2_O ratios (*z*) of 2.5 and 4.0 showed extensive gel formation. However, the matrix of the specimen with *z* = 2.5 was visibly denser and contained fine microcracks, likely due to drying shrinkage in a system with less free water. This denser microstructure is directly responsible for its substantially higher compressive strength compared to the more porous matrix formed at *z* = 4.0.

#### 3.8.2. MIP Analysis

**Figure 16:**
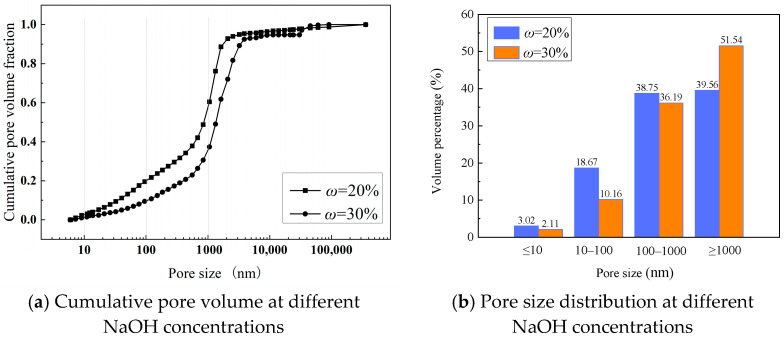

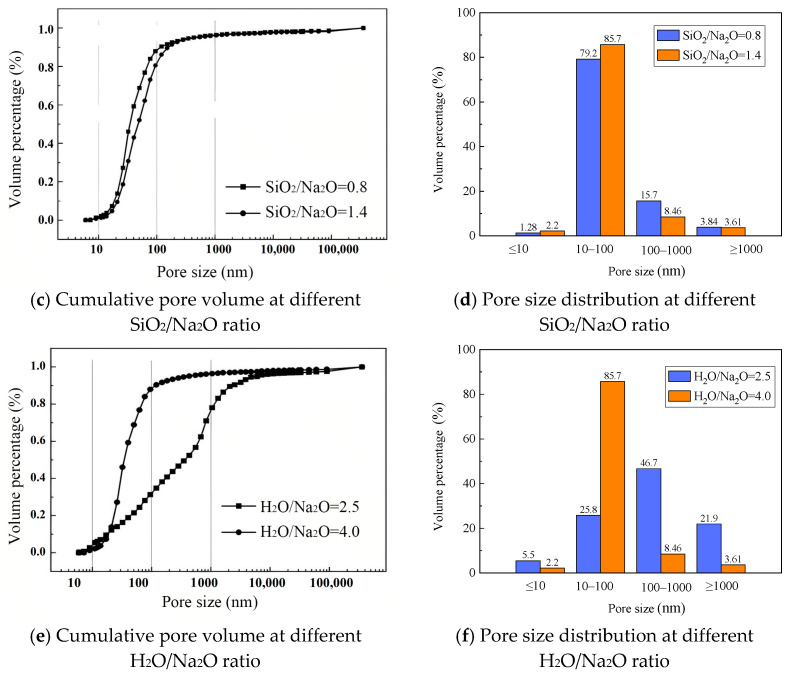
Cumulative pore volume and pore size distribution of mortar specimens.

The pore structure of the geopolymer mortars was quantified by MIP, with the cumulative pore volume curves and pore size distributions shown in Figure 16 and summarized in Table 5. The results corroborate the SEM observations and provide quantitative support for the mechanical property trends. Based on common classifications for cementitious materials, the pore structure is divided into four categories for analysis: gel pores (≤10 nm), transition pores (10~100 nm), capillary pores (100~1000 nm), and macropores (>1000 nm).

The influence of activator composition on the pore structure is multifaceted. Increasing the NaOH concentration from 20% to 30% reduced the total porosity from 21.4% to 19.7%. However, this was accompanied by a shift in the pore distribution towards larger pores, suggesting that while higher alkalinity promotes more FA dissolution, it may not necessarily lead to a more refined geopolymer gel, aligning with the strength results where an optimum concentration was observed.

The SiO_2_/Na_2_O ratio had a more pronounced effect on pore refinement. Although the total porosities for specimens with *n* = 0.8 and *n* = 1.4 were similar (21.0% and 19.8%), the latter exhibited a significant refinement of the pore structure. The average pore size decreased sharply from 112.2 nm to 42.5 nm, which was a result of a substantial increase in the volume of beneficial gel and transition pores at the expense of larger, more detrimental capillary pores. This pore refinement is a key factor contributing to the higher strength observed at the optimal SiO_2_/Na_2_O ratio.

The most dramatic effect on porosity was from the water content. Reducing the H_2_O/Na_2_O ratio (*z*) from 4.0 to 2.5 caused a significant decrease in total porosity from 19.8% to 14.4%. This reduction in overall void space, achieved by minimizing the volume of excess water that creates large capillary pores upon evaporation, is the primary reason for the substantial strength enhancement at lower water contents. In summary, the MIP results confirm that a high-strength geopolymer is characterized not only by low total porosity but, more importantly, by a refined pore structure dominated by smaller, less harmful pores.

## 4. Predictive Models for Mechanical Strength

### 4.1. Multivariate Regression Model for Mortar Strength

Based on the single-factor analyses, a multivariate generalized linear regression model was developed to predict the compressive strength (*f_c_*_,*M*_) and flexural strength (*f_f_*_,*M*_) of the geopolymer mortar as a function of the key influencing factors. The model considers linear relationships for the H_2_O/Na_2_O ratio (*z*), solution-to-binder ratio (*S*), and cement-to-sand ratio (*C*); a quadratic relationship for the SiO_2_/Na_2_O ratio (*n*) and curing temperature (*T*); a logarithmic relationship for curing duration (*t*); and linear/parabolic relationships for GGBFS content (*ω*). Excluding interaction effects, the resulting predictive equations are:

(11) $$f_{c,M}=-152.22-55.32n^{2}+133.73n-16.20z-0.03T^{2}+4.50T+9.74\ln(t)+4.72S-0.97C+67.72\omega$$

(12) $$f_{f,M}=4.72-9.791n^{2}+23.53n-2.20z-0.026T^{2}+0.49T+0.65\ln(t)-6.99S-1.96C-20.53\omega^{2}$$

The coefficient of determination (*R*^2^) for compressive strength is 0.915, and that for flexural strength is 0.856. These relatively high *R*^2^ values indicate that the proposed model provides a good fit to the experimental data.

### 4.2. Compressive Strength Prediction Model for Concrete Using Mortar Strength

To establish a predictive link between the properties of the mortar and the concrete, the size effect on mortar strength and the influence of coarse aggregate content were quantified.

#### 4.2.1. Size Effect on Mortar Compressive Strength

**Figure 17:**
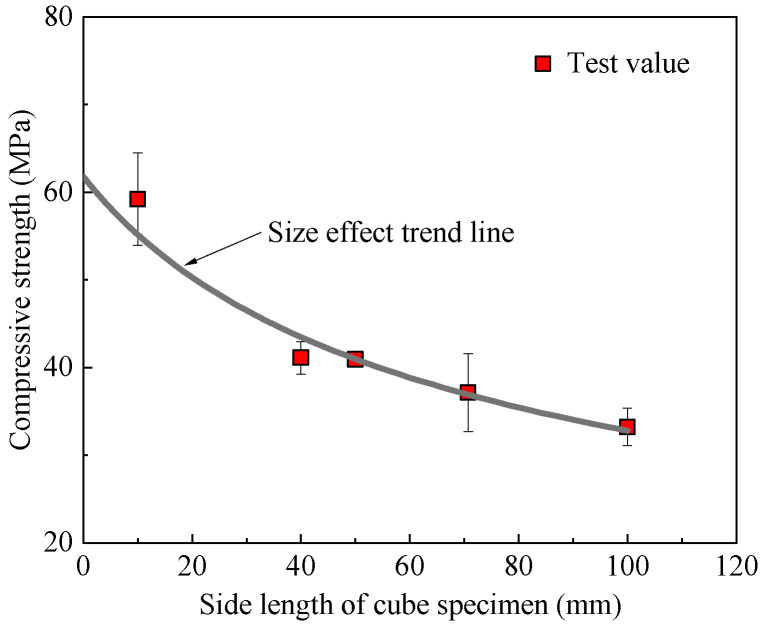
Size effect curve of geopolymer mortar specimens.

Cubic mortar specimens with side lengths (*D*) of 10, 40, 50, 70.7, and 100 mm were tested. As shown in Figure 17, the compressive strength decreased as the specimen size increased, a well-known size effect in quasi-brittle materials. Based on the classical statistical size effect theory, the relationship between compressive strength (*f_c_*_,C_) and specimen size (*D*) was fitted to the following equation:

(13) $$f_{c,C}=\frac{Bf_{t}^{\prime }}{\sqrt{1+\beta }},\;\beta =D/D_{0}$$

Therefore,

(14) $${\left(\frac{f_{t}^{\prime }}{f_{c,C}}\right)}^{2}=\frac{D}{B^{2}D_{0}}+\frac{1}{B^{2}}$$

Let $Y={\left(f_{t}^{\prime }/f_{c,C}\right)}^{2}$, X=D, $C=1/B^{2}$, $A=C/D_{0}$, it can be expressed as:

(15) Y=AX+C

In the equation, *B* and *D*_0_ are undetermined constants; $f_{t}^{\prime }$ is primarily used for unit adjustment and can be taken as the tensile strength, i.e., $f_{t}^{\prime }=1\;\mathrm{MPa}$. Regression analysis shows that *A* and *C* are 6.675 × 10^−6^ and 2.620 × 10^−4^, respectively, with a coefficient of determination (*R*^2^) of 0.935.

A size effect factor, $\alpha_{1}$, can be defined as the ratio of the strength of a 100 mm specimen to that of a 40 mm specimen (the size used for standard mortar testing):

(16) $$\alpha_{1}=\frac{100}{R_{c,40}\cdot \sqrt{0.06675\cdot D+2.620}}$$

#### 4.2.2. Effect of Coarse Aggregate Content

**Figure 18:**
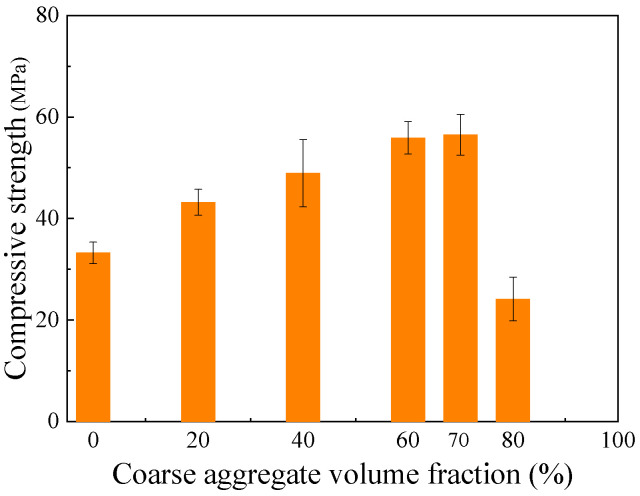
Effect of coarse aggregate fraction on compressive strength of geopolymer concrete.

The effect of coarse aggregate volume fraction on the compressive strength of geopolymer concrete is shown in Figure 18. The strength first increased with aggregate content, reaching a peak at approximately 60~70%, and then decreased. This is because a moderate number of aggregate forms a rigid skeleton that enhances stress transfer, while an excessive amount leads to poor compaction and increased defects. An aggregate content factor, α_2_, can be defined to capture this effect.

(17) $$\alpha_{2}=1+0.0815\sqrt{V}$$

In the equation, *V* is the coarse aggregate volume fraction, expressed as a percentage.

#### 4.2.3. Final Predictive Model and Validation

Assuming the absence of a distinct interfacial transition zone in geopolymer concrete, its compressive strength (*f_c_*_,C_) can be predicted based on the standard mortar compressive strength (*f_c_*_,M_), modified by the size effect factor (*α*_1_) and the coarse aggregate content factor (*α*_2_):

(18) $$f_{c,C}=f_{c,M}\cdot \alpha_{1}\cdot \alpha_{2}$$

**Figure 19:**
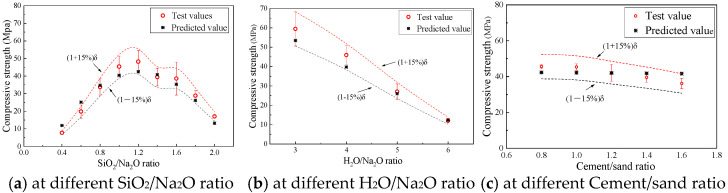
Predicted and experimental compressive strength of geopolymer concrete.

The model’s predictions were compared with experimental results for concrete specimens with varying mix proportions, as shown in Figure 19. The predicted trends for the effects of the SiO_2_/Na_2_O ratio, H_2_O/Na_2_O ratio, and cement/sand ratio are consistent with the experimental data. The relative error between the predicted and measured values was generally within 15%, indicating that the proposed model provides a reasonable estimation of geopolymer concrete strength based on mortar properties.

## 5. Conclusions

This study systematically investigated the influence of various material and curing parameters on the mechanical properties of geopolymer paste, mortar, and concrete. The failure mechanisms were elucidated through microstructural and pore structure analyses, leading to the development of predictive strength models. The key findings are as follows:

(1) The chemical characteristics of the activator and the curing temperature are the primary factors controlling the mechanical performance of geopolymers. Both compressive and flexural (or splitting tensile) strengths initially increase and then decrease with rising NaOH concentration or sodium silicate modulus, with optimal ranges of 24~26% for NaOH concentration and 1.2~1.4 for the SiO_2_/Na_2_O ratio. Compressive and flexural strengths decrease almost linearly with increasing H_2_O/Na_2_O ratio, indicating that the lower limit of this ratio is dictated by workability requirements. Thermal curing accelerates strength development and temperatures of 70~80 °C markedly enhance reaction rates. Although prolonging heat curing can further increase geopolymer strength, the efficiency gain is limited; therefore, a curing duration within 24 h is recommended.
(2) The mechanical strength of geopolymer mixtures is sensitive to mix proportions. For mortar, increasing the solution-to-binder ratio from 0.4 to 0.65 moderately reduces compressive strength and decreases flexural strength by 19.9%, indicating that the lowest ratio ensuring workability is preferable. An optimal binder-to-sand ratio also exists, as excessive binder can weaken tensile-related properties, while compressive strength remains relatively stable. For concrete, compressive strength increases with coarse aggregate content up to 60~70%, then declines. Careful selection of solution/binder, binder/sand, and aggregate ratios is therefore essential to optimize performance.
(3) SEM and MIP analyses indicate that at 20% NaOH concentration, a SiO_2_/Na_2_O ratio of 0.8, and H_2_O/Na_2_O of 4.0, SEM images frequently show residual unreacted particles accompanied by increased microcracking and interparticle debonding. In contrast, at 30% NaOH, a SiO_2_/Na_2_O ratio of 1.2, and H_2_O/Na_2_O of 2.5, SEM reveals a progressive reduction in identifiable unreacted fly ash particles and the formation of a more homogeneous and dense gel matrix. MIP data further corroborate these observations: under the latter conditions, total porosity decreases and pore throats are refined, whereas the former conditions lead to increased meso- and macroporosity or reduced connectivity of the gel network. Overall, superior mechanical performance of geopolymer materials corresponds to higher gel content, fewer unreacted particles, and lower porosity.
(4) Grey relational analysis ranks compressive strength influence as: curing temperature > silicate modulus > water content > liquid-to-solid ratio > binder-to-sand ratio > curing time > GGBFS content; flexural strength follows a similar trend. According to the regression analyses, all three material systems exhibited strong square-root-type correlations between compressive strength and flexural or splitting tensile strength. A generalized regression model was also developed to relate mortar strength to the compressive strength of geopolymer concrete, incorporating both size-effect and coarse-aggregate content corrections. The model further enables prediction of splitting tensile strength. At the current stage, partial validation indicates that the model can reliably capture the strength-variation trends of geopolymer concrete with respect to the SiO_2_/Na_2_O ratio, the H_2_O/Na_2_O ratio, and the binder-to-sand ratio.

These findings provide a quantitative framework for mix design and process optimization, enabling the production of high-performance, low-carbon geopolymer concrete with tailored strength characteristics. However, the study is limited by its use of a single FA source and tightly controlled curing conditions; variations in FA chemistry, long-term durability, shrinkage behavior, and environmental exposure were not fully examined. Future research should extend the proposed models to a broader range of raw materials and field conditions to enhance their general applicability.

## Figures and Tables

**Table 1:** Chemical composition and loss on ignition of FA and GGBFS.

Sample	SiO_2_	Al_2_O_3_	Fe_2_O_3_	CaO	MgO	Na_2_O	K_2_O	SO_3_	P_2_O_5_	TiO_2_	Others *	LOI **
Fly ash, %	59.87	24.66	4.14	3.84	1.23	0.45	0.18	0.71	0.30	1.27	0.15	3.20
GGBFS, %	33.20	15.00	0.81	35.07	6.34	0.39	0.61	2.43	0.00	2.34	1.51	2.30

* Others refers to the total amount of SrO, Cr_2_O_3_, MnO, ZnO, BaO, ZrO_2_, Rb_2_O and Y_2_O_3_. ** LOI denotes loss on ignition.

**Table 2:** The detailed mix proportions of geopolymer paste, mortar, and concrete.

Geopolymer Materials	Solution/Binder Ratio	Binder/Sand Ratio	Sand Ratio	*n*	*z*
Paste	0.4	-	-	1.0	4.0
Mortar	0.5	0.5	-	1.0	4.0
Concrete	0.5	0.6	33%	1.0	4.0

**Table 3:** Factors and corresponding levels investigated by the single-factor method.

Single-Factor	Variable Range	Number of Specimens
FA fineness (μm)	45–10.5, 10.5–4.3, ≤4.3	18 **
NaOH concentration (%)	12, 14, 16, 18, 20, 22, 24, 26	48 ** + 48 *
*n*	0.2, 0.4, 0.6, 0.8, 1.0, 1.2, 1.4, 1.6, 1.8, 2.0	60 ** + 60 *
*z*	2.0, 3.0, 4.0, 5.0, 6.0	30 ** + 24 *
*T* (°C)	40, 50, 60, 70, 80, 90	18 **
*t* (day)	1.0, 2.0, 3.0, 4.0, 5.0	15 **
*S*	0.4, 0.45, 0.5, 0.55, 0.6, 0.65	33 **
*C*	0.4, 0.5, 0.6, 0.7, 0.8; (0.8, 1.0, 1.2, 1.4, 1.6) *	15 ** + 30 *
*ω* (%)	12.5, 20, 40, 60, 80	15 **
*D* (mm)	10, 40, 50, 70.7, 100	15 **
*V* (%)	0.0, 20, 40, 60, 70, 80	18 *

Note: *T* = Curing temperature; *t* = Curing temperature; *S* ratio = mass ratio of activator solution to binder; *C* = mass ratio of binder to sand; *ω* = mass ratio of GGBFS to total binder; *V* = volume fraction of coarse aggregate; *D* = Edge length of the cube specimen; * indicates that the data apply to concrete; ** indicates that the data apply to mortar and paste.

**Table 4:** Grey correlation coefficients of influencing parameters.

Parameter	*n*	*z*	*T*	*t*	*s*	*c*	*ω*
Compressive Strength	0.8553	0.8531	0.8683	0.8044	0.8474	0.8447	0.8008
Flexural Strength	0.8862	0.8859	0.9040	0.8374	0.8816	0.8820	0.8278

**Table 5:** Pore structure parameters of geopolymer mortars.

Variable	NaOH Concentration		SiO_2_/Na_2_O Ratio		H_2_O/Na_2_O Ratio	
	20%	30%	0.8	1.4	2.5	4.0
Porosity	21.4%	19.7%	21.0%	19.8%	14.4%	19.8%
Average pore size	96.4 nm	153.8 nm	112.2 nm	42.5 nm	60.3 nm	32.6 nm

## Data Availability

The original contributions presented in this study are included in the article. Further inquiries can be directed to the corresponding author.

## Abbreviations

List of abbreviations in alphabetic order

Abbreviation	Meaning
CO_2_	Carbon dioxide
OPC	Ordinary Portland cement
FA	Fly ash
GGBFS	Ground granulated blast furnace slag
SiO_2_	Silicon dioxide
Al_2_O_3_	Aluminum oxide
NaOH	Sodium hydroxide
H_2_O	Water
Na_2_SiO_3_	Sodium silicate solution
*n*	Molar ratio of silicon dioxide to sodium oxide
*z*	Molar ratio of water to sodium oxide
*S*	Mass ratio of activator solution to binder
*C*	Mass ratio of binder to sand
*ω*	Mass ratio of GGBFS to total binder
*V*	Volume fraction of coarse aggregate
*T*	Curing temperature
*t*	Curing duration
*R*^2^	Coefficient of determination
GRA	Grey relational analysis
XRF	X-ray fluorescence
XRD	X-ray diffraction
D50	Median particle sizes
SEM	Scanning electron microscopy
MIP	Mercury intrusion porosimetry
